# A competency framework on simulation modelling-supported decision-making for Master of Public Health graduates

**DOI:** 10.1093/pubmed/fdad248

**Published:** 2023-12-07

**Authors:** Rok Hrzic, Maria Vitoria Cade, Brian Li Han Wong, Nicky McCreesh, Judit Simon, Katarzyna Czabanowska

**Affiliations:** Department of International Health, Care and Public Health Research Institute – CAPHRI, Maastricht University, Maastricht, 6200 MD, Netherlands; Department of International Health, Care and Public Health Research Institute – CAPHRI, Maastricht University, Maastricht, 6200 MD, Netherlands; Department of International Health, Care and Public Health Research Institute – CAPHRI, Maastricht University, Maastricht, 6200 MD, Netherlands; Department of Infectious Disease Epidemiology and Dynamics, Centre for Mathematical Modelling of Infectious Diseases, London School of Hygiene and Tropical Medicine, London, WC1E 7HT, UK; Department of Health Economics, Center for Public Health, Medical University of Vienna, Vienna, 1090, Austria; Department of International Health, Care and Public Health Research Institute – CAPHRI, Maastricht University, Maastricht, 6200 MD, Netherlands; Department of Health Policy Management, Institute of Public Health, Jagiellonian University, Krakow, 31-066, Poland

**Keywords:** education, employment and skills, health intelligence, models

## Abstract

**Background:**

Simulation models are increasingly important for supporting decision-making in public health. However, due to lack of training, many public health professionals remain unfamiliar with constructing simulation models and using their outputs for decision-making. This study contributes to filling this gap by developing a competency framework on simulation model-supported decision-making targeting Master of Public Health education.

**Methods:**

The study combined a literature review, a two-stage online Delphi survey and an online consensus workshop. A draft competency framework was developed based on 28 peer-reviewed publications. A two-stage online Delphi survey involving 15 experts was conducted to refine the framework. Finally, an online consensus workshop, including six experts, evaluated the competency framework and discussed its implementation.

**Results:**

The competency framework identified 20 competencies related to stakeholder engagement, problem definition, evidence identification, participatory system mapping, model creation and calibration and the interpretation and dissemination of model results. The expert evaluation recommended differentiating professional profiles and levels of expertise and synergizing with existing course contents to support its implementation.

**Conclusions:**

The competency framework developed in this study is instrumental to including simulation model-supported decision-making in public health training. Future research is required to differentiate expertise levels and develop implementation strategies.

## Introduction

Simulation models, frequently also called mathematical models,^1^ are computer-generated representations of a real-world system or process that can support decision-making in public health.^2^^,^^3^ Examples of use include projecting transmissions in an infectious disease outbreak, exploring the potential impact of different policy interventions and highlighting relationships between various services in the health system (see Table 1 for an overview). During the COVID-19 pandemic, simulation models were widely used to guide government policy.^4–7^

**Table 1:** Examples of the main modelling approaches in public health

Modelling approach	Description of the approach	Examples of its use in public health
Agent-based models	Model interactions between individuals (‘agents’) and between individuals and their environment. Each simulated individual can have unique characteristics, behaviours and reactions	Estimating the population health outcomes of delayed second dose versus standard schedule of SARS-CoV-2 mRNA vaccination^18^
Cohort and individual state transition models, microsimulation	Model individual or cohort lifepaths across time in different states, for example, disease progression, accounting for individual characteristics and current state in the transition probabilities. The states may be associated with costs, supporting economic evaluation	Assessing clinical and economic outcomes and cost-effectiveness of COVID-19 epidemic control strategies^19^
Compartmental models	Similar to state transition models, they model the transition of individuals between different disease stages (compartments), for example, susceptible, infected and recovered. The transitions can be modelled as a deterministic or a stochastic process. Simulated individuals within each compartment are usually assumed to be homogeneous	Exploring the potential impact of three social determinants of health on transmission dynamics and severity of COVID-19 across different countries^20^
System dynamics models	Model the behaviour of a complex system focusing on feedback loops, stocks and flows. Usually do not simulate the behaviours or states of individuals or specific cohorts	Exploring how health-seeking behaviour during pregnancy through to delivery affects neonatal outcomes^21^

This table presents a simplified overview of modelling archetypes, prioritizing representing the most common terminology clusters. There are important overlaps between these approaches, and they may be combined in single projects.

Despite their utility and increasing popularity in policymaking, simulation models and their use as decision aids are not a routine part of public health education.^8^ As a result, public health graduates are often not well trained in model construction and interpretation of their results. Unfamiliarity with common modelling assumptions, interpreting prediction errors and appropriately communicating modelling results has resulted in the occasional misuse of simulation modelling outputs in decision-making during the COVID-19 pandemic.^4^^,^^6^ It has also hindered the transdisciplinary collaboration required to develop appropriate models.^9^^,^^10^

A key factor that may have prevented simulation modelling from being included in routine public health training is a lack of a relevant competency framework. There is a need for a public health workforce proficient in systems thinking,^11^ and simulation modelling has long been viewed as a tool supporting it.^12^^,^^13^ However, previously developed competency frameworks have focussed on a specific modelling approach without explicitly considering the context of public health^14^ or have encompassed knowledge translation and systems thinking within public health without explicitly considering simulation modelling as a key tool in both endeavours.^8^^,^^15^^,^^16^

To our knowledge, no competency frameworks are currently aimed at public health professionals focussed on using simulation modelling to support decision-making. Whereas competency frameworks promote high standards in professional public health practice, support the professionalization of the public health workforce and guide the design of public health curricula,^15^^,^^17^ the lack of a competency framework in simulation modelling-supported decision-making hinders the effective development of public health workforce capacity in this direction.

The study develops a competency framework for simulation modelling-supported decision-making for Master of Public Health (MPH) students. It is intended to be broadly relevant across various modelling approaches (see Table 1). This competency framework can guide the design of educational interventions and the development of assessment tools for the students and professionals to identify their training needs and gaps in knowledge and skills. The framework’s users include teaching staff at schools of public health, students and recent graduates of MPH programmes and public health professionals interested in continuous professional development in decision-making.

## Methods

The study was carried out in three phases: a literature review, a two-stage online Delphi survey and an online consensus workshop. The report follows the CONFERD-HP reporting recommendations.^22^

### Literature review

We included peer-reviewed scientific articles, expert opinions or project reports published between 1 January 2010 and 1 February 2022, which discuss existing competency frameworks or explicitly discuss or identify competencies, best practices and recommendations relevant to the building or using simulation models for decision-making in public health and health policy. No language restrictions were imposed (see Supplementary material).

We searched the WoS Core, MEDLINE and ERIC scientific databases. We also screened the System Dynamics Society online bibliography database (https://systemdynamics.org/bibliography/) and websites of existing courses on dynamic simulation modelling and main simulation centres, namely:

- The Centre for the Mathematical Modelling of Infectious Diseases (CMMID) at LSHTM
- School of Health and Related Research at Sheffield University
- Complexity Science Hub Vienna

The keyword strategy focussed on four key concepts: simulation modelling, use for decision and policymaking, competencies and public health and health policy (see Supplementary material).

The selection was performed in two stages. First, titles and abstracts of identified records were screened for relevance. Second, the full text of retained records was examined against the inclusion and exclusion criteria. Two researchers (R.H. and M.V.C.) independently performed both stages. Differences were discussed until a consensus was reached.

From each included publication, the following data were extracted: publication reference (authors, year, title), study setting and relevant competencies. Competencies were identified in the included studies as explicit or implied descriptions of behaviours exemplifying relevant attitudes, knowledge or skills. Extracted competencies were deduplicated and organized in one of three phases of model development and use.

### Delphi survey

To refine the framework, a two-stage online Delphi survey^23^ was conducted between June 2022 and January 2023. In the first stage, a list of experts in simulation modelling-supported decision-making in public health and health policy was identified based on the results of the literature review and the recommendations by the project steering board. In the second stage, additional experts in health policy and decision-making, knowledge transfer in public health and public health education were identified by the steering board and invited to participate.

The Delphi surveys consisted of 15 questions asking the participants to rate the relevance of the proposed competencies using a five-point Likert scale, provide feedback on their formulation and suggest missing competencies.

The data were collected via Qualtrics, an online survey service. The quantitative data were analysed by calculating descriptive statistics, and the qualitative data were analysed by identifying common themes through inductive coding.

### Consensus workshop

An online consensus workshop was held in February 2023 to evaluate the final draft competency framework and discuss its implementation in education. The experts who previously participated in the Delphi survey and the project steering board members were invited to the workshop. During the workshop, the results of the Delphi survey and the final draft competency framework were presented. Then, the participants were asked to discuss the framework in small groups and report their conclusions. The workshop audio was recorded, transcribed and analysed by identifying the key themes of feedback.

### Ethics

Participants in the Delphi survey and consensus workshop were approached via email, informed of the purpose of this study and allowed to self-select to participate through a written informed consent form. The project was reviewed by the Research Ethics Committee of the Faculty of Health, Medicine and Life Sciences of Maastricht University (FHML-REC/2022/051).

### Developer and governance groups

R.H. and M.V.C. collected data for the competency framework development. B.L.H.W., N.M., J.S. and K.C. oversaw the project and collaborated with R.H. and M.V.C. to create the framework. Regular online meetings were held to review the data and its implications for successive framework drafts. The developers and governance groups had expertise in public health, including knowledge translation, infectious disease modelling, health economics and public health workforce development.

## Results

### Literature review

A total of 1518 records were identified during the database and website searches (see Supplementary material). After the two screening steps, we included 28 records in the data extraction and synthesis process. We excluded 1189 records due to a lack of relevance, 45 records due to not mentioning concrete knowledge, skills or attitudes about simulation modelling, 8 records due to not focusing on simulation modelling at all and 2 records due to narrowly focusing on the minutiae of a specific modelling step. Finally, we extracted and synthesized data from 28 articles (see Supplementary material).

The included articles highlighted two main themes: the modelling process and best practices in model development. These themes were used to construct a draft competency framework (see Table 2). Based on the included articles, we also developed two idealized profiles critical to the model construction and use (see Text Box 1).

**Text Box 1 The Modeller and Facilitator profiles**

*The Modeller*

The Modeller is a technical expert on the modelling methodologies and is charged with coding or otherwise technically constructing the dynamic simulation model. To fulfil this role, they: (1) apply one or more modelling approaches, such as transmission models or system dynamics models; (2) write computer code or use off-the-shelf modelling software; (3) understand and apply best practices in model calibration and validation; (4) apply systems thinking (i.e. systematically consider how the future outcomes depend on the interactions of relevant factors or variables) and (5) participate in the process of participatory simulation modelling (i.e. various stakeholders collaborating in model construction, calibration and evaluation), including sufficient communication skills to convey the characteristics, strengths and limitations of the models they build to non-experts.

*The Facilitator*

The Facilitator supports and guides the model-building team throughout the model-construction process, focusing on the representation and collaboration of various stakeholders, building the model and using the outputs to shape decision-making. To fulfil this role, they: (1) understand evidence-informed public health and health policy decision-making in their governance context, including the healthcare context, the relevant population and the health problem to co-create a systems map (i.e. a description of the key variables and the relationships between them); (2) understand the theory and practice of knowledge transfer; (3) apply communication and group facilitation skills; (4) apply systems thinking (see above) and (5) understand one or more modelling approaches, including the relevant model-building tools and calibration and validation procedures, well enough to facilitate collaboration with the Modeller.

**Table 2:** The development of the competency framework

Initial competency (identified in the literature)	Revised competency (after 1^st^ Delphi round)	Final competency (after second Delphi round and consensus workshop)	Comments
1.1 Demonstrates knowledge of basic business practices, such as terms of reference, business plans, contracting and project management	—	—	Deemed less relevant for this competency framework
1.2 Understands the principles of systems thinking	1.1 Understands the principles of systems thinking	1.1 Understands the principles of systems thinking	
1.3 Reflects on the strengths and weaknesses of different modelling methodologies	1.2 Reflects on the strengths and weaknesses of different modelling methodologies	1.2 Reflects on the strengths and weaknesses of different modelling methodologies and communicates the limits of modelling	Explicit mention of the limits of modelling
1.4 Identifies, connects and manages relationships with stakeholders	1.3 Identifies, connects and manages relationships with stakeholders throughout the model building process	1.3 Manages an accessible and equitable process of stakeholder participation throughout the model-building process	Emphasis on the importance of managing stakeholder relationships throughout the process
1.5 Builds consensus on the decision problem	1.4 Builds consensus on the decision problem	1.4 Builds consensus on the decision problem	
1.6 Builds consensus on the model boundaries	1.5 Builds consensus on the model boundaries	1.5 Builds consensus on the model boundaries	
1.7 Reflects on the role of participatory simulation modelling in diverse governance contexts	1.6 Reflects on the role of participatory simulation modelling in diverse governance contexts	1.6 Reflects on the role of participatory simulation modelling in diverse governance contexts	
2.1 Reflects on the principles of good dynamic simulation modelling	2.1 Reflects on the principles of good dynamic simulation modelling	2.1 Reflects on the principles of good dynamic simulation modelling	
2.2 Co-creates conceptual system maps^a^	2.2 Co-creates conceptual system maps	2.2 Co-creates conceptual system maps	
2.3 Knows how to retrieve, analyse and appraise evidence from all data sources to support decision-making	2.3 Knows how to retrieve, analyse and appraise evidence from all data sources to support decision-making	2.3 Knows how to retrieve, analyse and appraise evidence from various data sources to support decision-making	
2.4 Builds consensus on the key variables and relationships between the variables	2.4 Builds consensus on the key variables and relationships between the variables	2.4 Builds consensus on the key variables and relationships between the variables	
—	—	2.5 Utilizes modelling software or programming language	
—	—	2.6 Utilizes repositories of existing models and code	
2.5 Applies appropriate model calibration procedures	2.5 Applies appropriate model calibration procedures to account for potential biases and uncertainties in the input parameters and calibration targets	2.7 Applies appropriate calibration procedures to account for uncertainty in the input parameters and calibration targets	Explicit mention of uncertainty.
2.6 Applies appropriate sensitivity analysis procedures	2.6 Applies appropriate sensitivity analysis procedures to understand the effect of implicit and explicit model assumptions	2.8 Applies appropriate sensitivity analysis procedures to understand the effect of implicit and explicit model assumptions	Explicit mention of model assumptions
2.7 Applies appropriate validation procedures with a focus on historical fit	2.7 Applies appropriate validation procedures with a focus on historical fit	—	Merged with 2.7
2.8 Applies appropriate validation procedures with a focus on face validity	2.8 Applies appropriate validation procedures with a focus on face validity	—	Merged with 2.7
	2.9 Translates uncertainty of model outputs	2.9 Assesses and communicates the uncertainty and equity implications of model outputs	
3.1 Understands the process and aims of evidence-informed decision-making and knowledge transfer in diverse governance contexts	3.1 Understands the process and aims of evidence-informed decision-making and knowledge transfer in diverse governance contexts	3.1 Understands the process and aims of evidence-informed decision-making and knowledge transfer in diverse governance contexts	
3.2 Uses the model to evaluate different policy options	3.2 Uses the model to evaluate different policy options	3.2 Utilizes the model to evaluate different policy options	
3.3 Identifies the policy-implications of the model results	3.3 Identifies the policy-implications of the model results	3.3 Identifies the policy-implications of the model results	
3.4 Communicates the results effectively within the context of translating science and evidence into practice and policy	3.4 Communicates the results effectively within the context of translating science and evidence into practice and policy	3.4 Communicates the model results within the context of translating science and evidence into practice and policy	
—	3.5 Assesses when the use of the model beyond the stipulated boundaries (generalizability) is appropriate	3.5 Assesses model generalizability and reusability	
3.5 Enables the use of the model beyond the immediate decision problem	3.6 Enables the use of the model beyond the immediate decision problem if appropriate	—	Merged with 2.6 and 3.5

^a^We use system maps to broadly denote the descriptions of key variables and the relationships between them.

A key message in the literature was that successful simulation model generation and use for decision-making is a collaborative and iterative process that should involve expert modellers, decision-makers and public health practitioners with direct experience of the problem being modelled. The input of decision-makers and content-matter experts is especially critical to understanding the decision problem and conceptual modelling, which precede the technical task of model building.^24–26^ Clear communication and the development of trust between public health professionals and modellers are highlighted as supporting using modelling outputs in decision-making.^24^ The collaborative approach of model construction in health policy was referred to as participatory simulation modelling,^2^^,^^27–30^ which is analogous to group model building.^31^

Publications in the second theme focussed on best practices in specific model construction. They included recommendations for selecting a modelling approach, designing, calibrating and validating the model and using the model outputs.^32–39^ These can be seen in conversation with publications that reflect on the key challenges or common methodological shortcomings of published models.^9^^,^^40–44^ Good practice recommendations range from general principles to methodological prescriptions or solutions to specific modelling questions, for example, modelling the problem instead of the system, using models as tools for communication between disciplines, transparency in using evidence and assumptions in model calibration and the ease-of-use of the model and its outputs.^37^^,^^44^

### Delphi survey

#### Sample

The first round of the Delphi survey was concluded in August 2022. The sample consisted of four participants from Chile, England, Austria and the Netherlands with 5–30 years of experience in simulation modelling, including mathematical modelling, transmission modelling, microsimulation, agent-based modelling and system dynamics. All but one expert self-identified as also having expertise in public health.

The second round was concluded in February 2023. The sample consisted of 12 participants from the UK (*n* = 5), the Netherlands (*n* = 3), Portugal (*n* = 2), Greece and France with 5–30 years of experience building and using models, teaching simulation modelling, knowledge transfer, public health education and health policymaking.

#### Survey results

In the first survey round, the participants identified all but competencies 1.1 and 3.5 as relevant (mean score > 3.50, see Supplementary material). The participants also identified missing competencies about analytic thinking, model assumptions, performance and generalizability, input source bias and output uncertainty; stressed that stakeholder participation should be continuous and highlighted testing of multiple model structures to reduce structural uncertainty. The participants highlighted that both competency profiles are important for the modelling process and are often combined in a single person. Based on this feedback, we updated the draft competency framework (Table 2).

In the second survey round, all competencies received a mean relevance score of 3.45 (competency 1.5) or higher (see Supplementary material). The participants identified missing competencies related to equity and accessibility of the modelling process and outcomes, modelling software and code and model management and repositories. They reiterated the interdependence between the Modeller and Facilitator profiles (see Box 1).

### Consensus workshop

Six experts and four members of the development and governance groups participated in the consensus workshop. All experts also participated in at least one of the Delphi survey rounds and understood the overall study aim.

Participants primarily discussed competency profiles and implementing them in public health education. They suggested different phrasing for the Modeller and Facilitator profiles, arguing that the Modeller requires applied competencies related to Phase 2 while the Facilitator does not. They considered splitting the profiles into separate frameworks. Ultimately, all agreed on the importance of both profiles having some experience building models. Participants expressed doubt about a single course’s ability to cover competencies related to model construction, calibration and validation. They suggested clarifying the differences between the competencies of different levels of expertise. Finally, they highlighted that stakeholder management, use of evidence and knowledge translation competencies might already be covered in existing public health curricula, so synergies with existing modules could be found to support implementation.

The participants also suggested adding competencies related to communicating the limits of modelling, auditing models and modelling outputs and reusing existing models. They recommended minor adjustments to the formulation of competencies 2.5 and 2.7. Based on this feedback (see Table 2), the developer and governance group prepared a final version of the competency framework (see Table 3). The participants also reflected on the challenge of creating an overarching competency framework considering the often confusing and overlapping terminology regarding different types of modelling approaches. Finally, the participants noted that they would have liked the workshop to be longer as they felt they needed more time to reflect on all aspects of the competency framework.

**Table 3:** The final competency framework

Phase 1: project planning and stakeholder engagement	Phase 2: participatory model building and model calibration	Phase 3: consensus building for policy action
1.1 Understands the principles of systems thinking	2.1 Reflects on the principles of good dynamic simulation modelling	3.1 Understands the process and aims of evidence-informed decision-making and knowledge transfer in diverse governance contexts
1.2 Reflects on the strengths and weaknesses of different modelling methodologies and communicates the limits of modelling	2.2 Co-creates conceptual system maps^a^	3.2 Utilizes the model to evaluate different policy options
1.3 Manages an accessible and equitable process of stakeholder participation throughout the model-building process	2.3 Knows how to retrieve, analyse and appraise evidence from various data sources to support decision-making	3.3 Identifies the policy-implications of the model results
1.4 Builds consensus on the decision problem	2.4 Builds consensus on the key variables and relationships between the variables	3.4 Communicates the model results within the context of translating science and evidence into practice and policy
1.5 Builds consensus on the model boundaries	2.5 Utilizes modelling software or programming language	3.5 Assesses model generalizability and reusability
1.6 Reflects on the role of participatory simulation modelling in diverse governance contexts	2.6 Utilizes repositories of existing models and code	
	2.7 Applies appropriate calibration procedures to account for uncertainty in the input parameters and calibration targets	
	2.8 Applies appropriate sensitivity analysis procedures to understand the effect of implicit and explicit model assumptions	
	2.9 Assesses and communicates the uncertainty and equity implications of model outputs	

^a^We use system maps to broadly denote the descriptions of key variables and the relationships between them.

## Discussion

### Main finding of this study

We developed a framework of 20 competencies on simulation modelling-supported decision-making for MPH graduates using a literature review, a two-stage online Delphi survey and an online consensus workshop. The included competencies highlight the commonly observed professional roles of the modeller and the modelling process facilitator. The framework received support from participants in two Delphi survey rounds and an online consensus workshop. The concerns raised during the consultations include accounting for the differences in the professional roles of Modeller and Facilitator, integrating the identified competencies into existing public health curricula and identifying the appropriate level of expertise for each competency.

### What is already known on this topic

The COVID-19 pandemic brought simulation models and their impact on health policymaking into the limelight.^4–7^ However, they are not a routine part of public health training.^8^ The lack of a relevant competency framework is likely a critical reason for this omission. This study draws on previously developed competency frameworks, including frameworks in system dynamics modelling outside the context of public health^14^ and frameworks in knowledge translation and systems thinking in public health outside the context of mathematical modelling.^8^^,^^15^^,^^16^

### What this study adds

This study is the first to develop a competency framework related to simulation modelling and its use for decision-making in public health. The competency framework developed in this study synthesizes existing knowledge on simulation modelling in public health and operationalizes it for graduate-level public health education. This is a significant contribution because it simplifies the implementation of this topic in public health curricula while maintaining a link with competency development in other areas of public health and beyond, thereby supporting an interdisciplinary perspective essential to public health workforce development.^45^

The key outstanding challenges are representing two idealized profiles of Modeller and Facilitator within the competency framework and differentiating levels of expertise. First, while separating these ideal profiles enhances clarity, participants in this study highlighted that the roles of Modeller and Facilitator are often combined in a single person and significantly overlap in their competencies. We believe an integrated competency framework is more appropriate for informing general graduate-level public health education. By contrast, a more specialized framework focussed on one of the profiles may be better suited for post-graduate specialization. Second, while the current study aimed to formulate competencies for a recent MPH graduate level, we concur with the need to extend the present work to encompass more advanced levels of expertise. This would significantly enhance the utility of the present competency framework by including postgraduate training and continuous professional development. For example, the World Health Organization–ASPHER Competency Framework for the Public Health Workforce in the European Region^8^ uses the Dreyfus model^46^ to identify three levels of expertise within each competency. We encourage future research in this direction.

After the experience of the COVID-19 pandemic, there is mounting pressure to emphasize new topics in public health curricula, for example, cultural competence^47^ and digitalisation.^48^ This may raise concerns about how to fit all this content into already packed MPH curricula. As highlighted by our respondents, an important solution is finding synergies with existing courses. For example, our framework includes competencies in stakeholder management (1.3), finding and appraising evidence (2.3) and evidence-informed decision-making (3.1), which are—to various extents—already covered by existing curricula. Furthermore, competencies 1.4, 1.5, 2.2 and 2.4, for example, could all be taught as practical elements of a course on stakeholder management, while competencies 2.5–2.8 could all find purchase in existing statistical modelling courses. While significant adaptations to existing MPH courses and curricula will be required, implementing the present competency framework may not require increasing the length of MPH education or relegating these critical skills to optional courses. Implementing this framework should be supported by research on the level of expertise in these competencies by recent graduates of MPH programmes. Insights from these studies can identify existing courses most relevant to the competencies we identified and the gaps that must be addressed by new educational material, as well as support the differentiation of levels of expertise discussed above.

### Limitations of this study

The study has several limitations. First, despite our best efforts, there may be implied competencies in the included studies that we did not include in the initial draft of the competency framework. For this reason, the Delphi survey also included questions prompting the participating experts to identify further relevant competencies. Second, the number of participating experts was limited. However, the high levels of agreement between the participants across the Delphi surveys and the consensus workshop indicate that the results are reliable. While some relevant contributors may have been excluded, we do not believe the limited samples significantly affected the outcomes. Third, the consensus workshop was perceived as too short by the participants. However, since most workshop participants were exposed to the same material in at least one prior survey round of consultation and the issues raised in the workshop were primarily relevant to implementation and expertise levels, we believe that the quality and depth of the outcomes were not significantly constrained. Fourth, the competency framework does not include highly detailed competencies that may be relevant for a senior expert in the Facilitator or Modeller role. However, this is aligned with the aims of this study and, in our view, enhances the likelihood it will be implemented into MPH curricula. Finally, we note that most literature and experts included are from the global north-west. We recommend that the users of our framework consider their national or local contexts during implementation.

## Supplementary Material

Simulation_modelling_decision-making_competency_framework_R1-supplementary_material_fdad248

## Data Availability

Anonymised datasets are available upon request.

## Notes

**Rok Hrzic,** Lecturer and Researcher

**Maria Vitoria Cade,** Research Assistant

**Brian Li Han Wong,** PhD Student

**Nicky McCreesh,** Researcher

**Judit Simon,** Professor

**Katarzyna Czabanowska,** Professor, Visiting Professor
