# Supplementary material for: A competency framework on simulation modelling-supported decision-making for Master of Public Health graduates
Source: J Public Health (Oxf). 2023 Dec 7;46(1):127–35. doi: 10.1093/pubmed/fdad248 (PMC10901273; doi:10.1093/pubmed/fdad248)
Supplement: Simulation_modelling_decision-making_competency_framework_R1-supplementary_material_fdad248 [file simulation_modelling_decision-making_competency_framework_r1-supplementary_material_fdad248.docx]

A competency framework on simulation-modelling-supported decision-making for Master of Public Health graduates

# Supplementary material

**Supplementary Table 1. Eligibility criteria**

| Inclusion criteria | Exclusion criteria |
| --- | --- |
| Published after January 1^st^, 2010  Peer-reviewed publications, expert opinions, and project reports  Publications that explicitly discuss or identify competencies, best practices, and recommendations relevant to the building or using simulation models for decision-making in public health and health policy  Any language | Published before January 1^st^, 2010  Technical and software manuals, conference proceedings  Publications narrowly discussing a particular facet of a specific modelling approach or technical details most relevant to senior experts (PhD-level and above) |

**Supplementary Table 2. Search strategy**

| **Simulation modelling** | **Use for decision and policy making** | **Competencies** | **Fields of public health and health policy** |
| --- | --- | --- | --- |
| “simulation model*”  OR  “mathematical model*”  OR  “dynamic model*”  OR  “infectious disease model*”  OR  “system dynamics”  OR  “complexity science”  OR  “systems science”  OR  “agent-based model*”  OR  ABM*  OR  Microsimulation*  OR  “computer simulation”  OR  “computer simulation”[Mesh][DE]  OR  “computer model*”  OR  “discrete event simulation*”  OR DES  OR  “monte carlo simulation*” | decision-mak*  OR  "decision mak*"  OR  “Decision making”[DE]  OR  Policy-mak*  OR  “policy mak*”  OR  “Policy making”[Mesh]  OR  Judg*  OR  Govern*  OR  Governance[DE]  OR  Manag* | competenc*  OR  Competence[DE]  OR  knowledge  OR  skills  OR  attitudes  OR  values  OR  Traits  OR  "Education, Public Health Professional"[Mesh]  OR  "Competency-Based Education"[Mesh]  OR  "Competency-Based Education" [DE] | "health policy"  OR  “Health Policy”[Mesh]  OR  “public health"  OR  "Public Health"[Mesh]  OR  “Public health” [DE]  OR  "population health"  OR  "Population Health"[Mesh]  OR  “health economics”  OR  Epidemiology  OR  Epidemiology [DE] |

**Supplementary Table 2. Studies included in the data extraction and synthesis**

| **Author(s)** | **Year** | **Study setting** | **Extracted competencies** |
| --- | --- | --- | --- |
| Chilcott, J., et al. | 2010 | Identification of challenges associated with HTA modelling and strategies to reduce those errors. | - Model validation - Qualitative analysis - Model validity analysis - Verification of computer model - Trust building - Face validity checking - Check for reasonable results - Black-box checking - Internal consistency analysis - Model input values analysis - Double-programming |
| Ainsworth, J. D., et al. | 2011 | Tool for creating, executing and analysing the results of a health model for interventions | - Transparency - Development of a versatile and flexible model - Focus on accessibility - Collaboration - Data management - Model validation |
| Prieto, D. M., et al. | 2012 | Identification of challenges associated with the practical implementation of the pandemic influenza models through survey | - Identifying data sources - Use of appropriate data estimators - Statistical validation - Understanding of social behaviours - Establish model feasibility by minimising running time and number of replicates |
| Tappenden, P., et al. | 2012 | Methodological framework on how to build 'Whole Disease Modelling' | - Definition of model boundary, breadth, and depth; - Understanding the decision problem; - Model conceptualisation, design and calibration; - Implementation of the modelling; - Model checking and evaluation, - Engaging with the decision. |
| Driedger, S. M., et al. | 2014 | Interviews about experiences of modellers and senior public health professionals about the usefulness of modelling during the pandemic H1N1 | - Convergent interview expertise - Public health priorities knowledge; - Clear communication and plain language - Definition of model boundaries; - Development of strong working relationships through collaboration and integration. |
| Macgillivray, B. H.; Richards, K. | 2015 | How model and governance types are interlinked | - Understanding of the different types of models - Knowledge of model structures and practices - Definition of boundaries and parameters - Computer coding - Assessment of dimensionality and resolution - Transparency - Knowledge of model-building methodology - Quality analysis through statistics - Knowledge of different governance types - Use of participatory approach |
| Marshall, D. A., et al. | 2015 | Comparison of different dynamic simulation models | - System dynamics knowledge - ABM knowledge - DES knowledge - Understand the benefits and disadvantages of different model types. - Definition of purpose and scope - Methodology design - Identify the different factors in the modelling - Identify data source and availability - Verify the quality of the model - Model calibration - Output and sensitivity analysis - Continuum reporting and transparency - Model maintenance and upkeep - Consensus of stakeholders - Use of SIMULATE checklist - Use of participatory approach |
| Moghadas, S. M., et al. | 2015 | Workshop building for simulation modelling of infectious disease in Canada | - Knowledge of infectious disease development - Communication - Stakeholder collaboration - Assessment of data quality and access - Definition of scope and purpose - Definition of roles and responsibilities - Identification of modelling capacity - Assessment of cost-effectiveness - Use of common language |
| Freebairn, L., et al. | 2016 | Application and evaluation of gestational diabetes simulation modelling | - Identifying data sources; - Parameterising the model; - Incorporating local and/or national data; - Identifying potential intervention leverage points; - Mapping the mechanism for model effect; - Knowledge of face validity, system behaviour reproduction, parameter estimation, sensitivity analysis and statistical testing; - Definition of purpose, assumptions and limitations of the model; - Optimising the model through timing, frequency and combination of interventions; - Participatory model building; - Incorporation of agent-based modelling components; - Planning of healthcare interventions - Evaluation of participatory approach |
| Northridge, M. E.; Metcalf, S. S. | 2016 | How to better approach modelling | - Modelling of the problem - Knowledge of both qualitative and quantitative data - Knowledge of boundary objects - Construction of a portfolio model |
| Schaffernicht, M. F. G.; Groesser, S. N. | 2016 | Best principles of system sciences for dissemination and application | - Definition of the problem, boundaries and limits - Map conceptualisation of the problem - Knowledge of qualitative and quantitative factors - Specification of equations and algorithms - Identification of logical behaviour boundaries - Sensitivity analysis - Identify boundary objects - Make of modelling portfolio - Trust building |
| Atkinson, J.-A., et al. | 2017 | Dynamic simulation model of possible policy actions to reduce alcohol-related harms in New South Wales (NSW), Australia (2015) | - Participatory model building and model validation - Project planning and engagement - Definition of purpose, scope and boundaries of the modelling project - Consensus building between stakeholders for policy actions - Making of modelling workshops - Expert technical advice - Dynamic simulation modeller - Data analysis - Logistical arrangements - Conceptual mapping - Documenting the steps to provide transparency in model structure, parameterisation and assumptions; |
| Freebairn, L., et al. | 2017 | Implementation of participatory approach in an Australian modelling setting | - Use of participatory approach - Definition of model scope and purpose - Model design and parameterisation - Identify key outputs and interventions to be tested and included - Identify and invite stakeholders with different views and approaches - Conceptual mapping - Workshop building - Consensus - Establishing effective partnerships - Use co-production methods - Trust building |
| Russell, R. E., et al. | 2017 | How to build a predictive model of infectious diseases | - Understanding the modelling frame and deciding which one is the most appropriate; - Defining which parameters or factors are critical for making preliminary predictions; - Collecting existing data to parameterise the initial models. |
| Currie, D. J., et al. | 2018 | Review identifying how system dynamics is being used to inform decision-making processes related to environmental health | - Use of participatory approach is needed, involving the stakeholders affected by or involved in decision-making processes - Understanding that the limitations are related to application and not to the model themselves:   1) user related; 2) technical; 3) application-related |
| Freebairn, L., et al. | 2018 | Perceptions of the value and efficacy of end-user decision-makers in participatory simulation modelling: case studies of the Australian health policy | - Use of participatory approach; - Trust building; - Communication; |
| Den Boon, S., et al. | 2019 | Review providing guidelines to provide for comparing different infectious disease models | - Expertise - Transparency - Definition of research question - Definition of timeline - Model design - Target definition - Data review - Bias minimisation - Quality assessment - Organisation and documentation - Harmonisation of parameters - Comparison of settings - Model variability exploration - Use of sensitivity analysis - Discussion of results |
| Freebairn, L., et al. | 2019 | Transforming system maps into models: how to use qualitative parameters | - Utilising storytelling for communication of results - Participatory approach needed for:  1. Definition of scope; 2) maximisation of utility |
| Porgo, T. V., et al. | 2019 | Common terminology about mathematical modelling in health | - Understand approaches - Understand purposes - Make use of the glossary - Understand different models - Understand model development - Understand model calibration and validation - Research question definition - Assessment on model suitability - Consider model uncertainty |
| Xie, T., et al. | 2019 | How to construct and improve parallel simulation decision-making models | - Knowledge of DES theory - Identifying the input-output-precondition-effect relationships - Defining the relationship flows - Designing of initial simulation - Knowledge of RP optimisation - Statistical analysis - Simulation execution - Knowledge of parallel simulation |
| Kretzschmar, M. | 2020 | Application, challenges and use of different disease modelling | - Find a suitable budgeting - Find suitable infrastructure - Find suitable data - Computation knowledge - Up-to-date technology knowledge - Knowledge of systems dynamics - Organising an internal peer review group - Communication - Model maintenance - Structural support of different modelling activities - Recruitment strategy |
| Brozek, J. L., et al. | 2021 | Guideline for assessing, developing and evaluating quantitative modelling (GRADE framework) | - Use of common glossary - Problem conceptualisation - Assess how to incorporate existing model outputs - Use of systematic review - Model calibration - Model validation - Model design - Assess model credibility - Bias minimisation - Assess indirectness - Sensitivity analysis - Data assessment - Use of dose-response gradient - Criteria development of indirectness - Variability analysis |
| Larrain, N.; Groene, O. | 2021 | Systematic review for performance assessment | - Knowledge of different modelling techniques - Understand the strengths and limitations of different modelling techniques - Understand the complexity of different modelling techniques - Model optimisation - Long term assessment |
| Mertens, E., et al. | 2021 | Review of microsimulation methodology | - Definition of purpose - Knowledge of different modelling approaches - Populate the model with synthetic individuals - Conceptualise individual risk factors and demographic parameters - Examine validity, sensitivity and parameter uncertainty |
| Adams, S., et al. | 2021 | How to use a participatory approach in health modelling | - Use of participatory approach - Definition of the problem - Reduction of conflict - Trust building - Promotion of acceptance and ownership |
| Almeda, N., et al. | 2022 | Impact of a specific policy designed to balance inpatient and community-based care provision by using the Bayesian network integrating the expert knowledge and statistical approach. | - Knowledge of modelling scenarios; - Conceptual mapping consensus; - Standard regression knowledge; - Knowledge of Monte Carlo simulation for intervention assessment; - Knowledge of computer-based Decision Support System (DSS); - Knowledge of RTE, statistical stability and entropy |
| Kretzschmar, M. E., et al. | 2022 | How to overcome challenges of modelling human-to-human transmission in pandemics | - Describe the system in sufficient detail - Simplify the model for use - Definition of purpose and aims - Design data collection study - Design statistical methodology - Minimise bias - Develop flexible models - Understanding of temporal and spatial scale of interventions - Modelling of population heterogeneity - Define and model the concept of elimination - Understanding of dynamic environments - Understanding and modelling of clusters - Parameterising behaviour factors - Parameterising interventions - Identification of intervention impacts statistics - Knowledge of health economics - Parameterising macroeconomic costs - Parameterising financial constraints - Parameterising inequalities |
| Schaffernicht, M.; Madariaga, P. | 2022 | Framework to identify the competencies of learners, from beginner to master learning | - Knowledge of Bloom’s taxonomy - Understanding of system dynamics language - Good reasoning - Analysis of sensitivity - Model validation - Definition of boundaries and purpose - Parameterisation - Policy evaluation - Policy design - Modelling project management |

**Identification of studies via databases and registers**

**Identification of studies via other methods**

Records identified from websites (n = 8)

Records removed *before screening*:

Duplicate records removed (n = 242)

Records excluded (n = 1189)

Reports assessed for eligibility

(n = 4)

Reports sought for retrieval

(n = 8)

Reports not retrieved

(n = 0)

Reports excluded:

Not focused on concrete competencies (n = 44)

Not focused on simulation modelling (n = 8)

Overly narrow focus (n = 2)

Records identified from databases (n = 1510):

ERIC (n = 5)

MEDLINE (n = 1065)

WoS Core (n = 440)

**Identification**

Records screened (n = 1268)

Reports included in review

(n = 28)

Reports sought for retrieval

(n = 79)

Reports assessed for eligibility

(n = 79)

Reports not retrieved

(n = 4)

**Screening**

Reports excluded:

Not focused on concrete competencies (n = 1)

**Included**

**Supplementary Figure 1. PRISMA flow diagram**

**Supplementary Table 3 Quantitative analysis of the general relevance of the competencies – 1^st^ round.**

| Question | Minimum | Maximum | Mean | Std Deviation | Count |
| --- | --- | --- | --- | --- | --- |
| 1.1 Demonstrates knowledge of basic business practices, such as terms of reference, business plans, contracting and project management | 1.00 | 2.00 | 1.75 | 0.43 | 4 |
| 1.2 Understands the principles of systems thinking | 4.00 | 5.00 | 4.50 | 0.50 | 4 |
| 1.3 Reflects on the strengths and weaknesses of different modelling methodologies | 4.00 | 5.00 | 4.75 | 0.43 | 4 |
| 1.4 Identifies, connects and manages relationships with stakeholders | 3.00 | 4.00 | 3.75 | 0.43 | 4 |
| 1.5 Builds consensus on the decision problem | 2.00 | 5.00 | 3.50 | 1.12 | 4 |
| 1.6 Builds consensus on the model boundaries | 4.00 | 5.00 | 4.50 | 0.50 | 4 |
| 1.7 Reflects on the role of participatory simulation modelling in diverse governance contexts | 3.00 | 5.00 | 4.00 | 0.71 | 4 |
| 2.1 Reflects on the principles of good dynamic simulation modelling | 4.00 | 5.00 | 4.75 | 0.43 | 4 |
| 2.2 Co-creates conceptual system maps | 2.00 | 5.00 | 3.50 | 1.12 | 4 |
| 2.3 Knows how to retrieve, analyse and appraise evidence from all data sources to support decision-making | 4.00 | 5.00 | 4.50 | 0.50 | 4 |
| 2.4 Builds consensus on the key variables and relationships between the variables | 3.00 | 4.00 | 3.75 | 0.43 | 4 |
| 2.5 Applies appropriate model calibration procedures | 4.00 | 5.00 | 4.50 | 0.50 | 4 |
| 2.6 Applies appropriate sensitivity analysis procedures | 4.00 | 5.00 | 4.50 | 0.50 | 4 |
| 2.7 Applies appropriate validation procedures with a focus on historical fit | 3.00 | 5.00 | 4.25 | 0.83 | 4 |
| 2.8 Applies appropriate validation procedures with a focus on face validity | 3.00 | 5.00 | 4.25 | 0.83 | 4 |
| 3.1 Understands the process and aims of evidence-informed decision-making and knowledge transfer in diverse governance contexts | 3.00 | 4.00 | 3.50 | 0.50 | 4 |
| 3.2 Uses the model to evaluate different policy options | 4.00 | 5.00 | 4.75 | 0.43 | 4 |
| 3.3 Identifies the policy implications of the model results | 4.00 | 5.00 | 4.50 | 0.50 | 4 |
| 3.4 Communicates the results effectively within the context of translating science and evidence into practice and policy | 4.00 | 5.00 | 4.75 | 0.43 | 4 |
| 3.5 Enables the use of the model beyond the immediate decision problem | 2.00 | 4.00 | 3.00 | 0.71 | 4 |

**Supplementary Table 4 Quantitative analysis of the general relevance of the competencies – 2^nd^ round.**

| Competency | Minimum | Maximum | Mean | Std Deviation | Count |
| --- | --- | --- | --- | --- | --- |
| 1.1 Understands the principles of systems thinking | 3.00 | 5.00 | 4.45 | 0.78 | 11 |
| 1.2 Reflects on the strengths and weaknesses of different modelling methodologies | 2.00 | 5.00 | 3.82 | 0.94 | 11 |
| 1.3 Identifies, connects and manages relationships with stakeholders throughout the process | 2.00 | 5.00 | 4.00 | 1.00 | 12 |
| 1.4 Builds consensus on the decision problem | 2.00 | 5.00 | 4.36 | 0.88 | 11 |
| 1.5 Builds consensus on the model boundaries | 4.00 | 5.00 | 4.45 | 0.50 | 11 |
| 1.6 Reflects on the role of participatory simulation modelling in diverse governance contexts | 2.00 | 5.00 | 3.45 | 0.89 | 11 |
| 1.7 Applies good analytical and critical thinking | 4.00 | 5.00 | 4.82 | 0.39 | 11 |
| 2.1 Reflects on the principles of good dynamic simulation modelling | 3.00 | 5.00 | 4.00 | 0.60 | 11 |
| 2.2 Co-creates conceptual system maps | 2.00 | 5.00 | 4.00 | 0.95 | 11 |
| 2.3 Knows how to retrieve, analyse and appraise evidence from all data sources to support decision-making | 2.00 | 5.00 | 4.18 | 0.94 | 11 |
| 2.4 Builds consensus on the key variables and relationships between the variables | 2.00 | 5.00 | 4.09 | 1.16 | 11 |
| 2.5 Applies appropriate model calibration procedures to account for potential biases and uncertainties in the input parameters and calibration targets | 2.00 | 5.00 | 3.73 | 0.86 | 11 |
| 2.6 Applies appropriate sensitivity analysis procedures to understand the effect of implicit and explicit model assumptions | 2.00 | 5.00 | 3.91 | 0.90 | 11 |
| 2.7 Applies appropriate validation procedures with a focus on historical fit | 2.00 | 5.00 | 3.91 | 1.00 | 11 |
| 2.8 Applies appropriate validation procedures with a focus on face validity | 2.00 | 5.00 | 4.09 | 1.00 | 11 |
| 2.9 Translates uncertainty of model outputs | 2.00 | 5.00 | 4.45 | 0.99 | 11 |
| 3.1 Understands the process and aims of evidence-informed decision-making and knowledge transfer in diverse governance contexts | 3.00 | 5.00 | 4.09 | 0.67 | 11 |
| 3.2 Uses the model to evaluate different policy options | 3.00 | 5.00 | 4.45 | 0.78 | 11 |
| 3.3 Identifies the policy implications of the model results | 4.00 | 5.00 | 4.64 | 0.48 | 11 |
| 3.4 Communicates the results effectively within the context of translating science and evidence into practice and policy | 4.00 | 5.00 | 4.64 | 0.48 | 11 |
| 3.5 Assesses when the use of the model beyond the stipulated boundaries (generalisability) is appropriate | 2.00 | 5.00 | 3.91 | 1.00 | 11 |
| 3.6 Enables the use of the model beyond the immediate decision problem if appropriate | 2.00 | 5.00 | 3.55 | 0.99 | 11 |

**Supplementary Table 5 Quantitative analysis of the relevance of the competencies for the Modeler profile – 1^st^ round.**

| Competency | Minimum | Maximum | Mean | Std Deviation | Count |
| --- | --- | --- | --- | --- | --- |
| 1.1 Demonstrates knowledge of basic business practices, such as terms of reference, business plans, contracting and project management | 1.00 | 4.00 | 2.25 | 1.09 | 4 |
| 1.2 Understands the principles of systems thinking | 5.00 | 5.00 | 5.00 | 0.00 | 4 |
| 1.3 Reflects on the strengths and weaknesses of different modelling methodologies | 5.00 | 5.00 | 5.00 | 0.00 | 4 |
| 1.4 Identifies, connects and manages relationships with stakeholders | 2.00 | 4.00 | 2.75 | 0.83 | 4 |
| 1.5 Builds consensus on the decision problem | 3.00 | 5.00 | 4.00 | 0.71 | 4 |
| 1.6 Builds consensus on the model boundaries | 4.00 | 5.00 | 4.50 | 0.50 | 4 |
| 1.7 Reflects on the role of participatory simulation modelling in diverse governance contexts | 2.00 | 4.00 | 2.75 | 0.83 | 4 |
| 2.1 Reflects on the principles of good dynamic simulation modelling | 5.00 | 5.00 | 5.00 | 0.00 | 4 |
| 2.2 Co-creates conceptual system maps | 2.00 | 5.00 | 4.00 | 1.22 | 4 |
| 2.3 Knows how to retrieve, analyse and appraise evidence from all data sources to support decision-making | 5.00 | 5.00 | 5.00 | 0.00 | 4 |
| 2.4 Builds consensus on the key variables and relationships between the variables | 3.00 | 5.00 | 4.50 | 0.87 | 4 |
| 2.5 Applies appropriate model calibration procedures | 5.00 | 5.00 | 5.00 | 0.00 | 4 |
| 2.6 Applies appropriate sensitivity analysis procedures | 5.00 | 5.00 | 5.00 | 0.00 | 4 |
| 2.7 Applies appropriate validation procedures with a focus on historical fit | 4.00 | 5.00 | 4.75 | 0.43 | 4 |
| 2.8 Applies appropriate validation procedures with a focus on face validity | 4.00 | 5.00 | 4.75 | 0.43 | 4 |
| 3.1 Understands the process and aims of evidence-informed decision-making and knowledge transfer in diverse governance contexts | 2.00 | 5.00 | 3.50 | 1.12 | 4 |
| 3.2 Uses the model to evaluate different policy options | 4.00 | 5.00 | 4.75 | 0.43 | 4 |
| 3.3 Identifies the policy implications of the model results | 2.00 | 5.00 | 3.75 | 1.09 | 4 |
| 3.4 Communicates the results effectively within the context of translating science and evidence into practice and policy | 2.00 | 5.00 | 3.75 | 1.09 | 4 |
| 3.5 Enables the use of the model beyond the immediate decision problem | 2.00 | 5.00 | 3.25 | 1.30 | 4 |

**Supplementary Table 6 Quantitative analysis of the relevance of the competencies for the Modeler profile – 2^nd^ round.**

| Competency | Minimum | Maximum | Mean | Std Deviation | Count |
| --- | --- | --- | --- | --- | --- |
| 1.1 Understands the principles of systems thinking | 3.00 | 5.00 | 4.44 | 0.83 | 9 |
| 1.2 Reflects on the strengths and weaknesses of different modelling methodologies | 2.00 | 5.00 | 4.44 | 1.07 | 9 |
| 1.3 Identifies, connects and manages relationships with stakeholders throughout the process | 2.00 | 5.00 | 3.44 | 1.26 | 9 |
| 1.4 Builds consensus on the decision problem | 1.00 | 5.00 | 3.56 | 1.17 | 9 |
| 1.5 Builds consensus on the model boundaries | 2.00 | 5.00 | 4.00 | 0.94 | 9 |
| 1.6 Reflects on the role of participatory simulation modelling in diverse governance contexts | 1.00 | 5.00 | 3.22 | 1.31 | 9 |
| 1.7 Applies good analytical and critical thinking | 4.00 | 5.00 | 4.78 | 0.42 | 9 |
| 2.1 Reflects on the principles of good dynamic simulation modelling | 3.00 | 5.00 | 4.78 | 0.63 | 9 |
| 2.2 Co-creates conceptual system maps | 3.00 | 5.00 | 4.22 | 0.79 | 9 |
| 2.3 Knows how to retrieve, analyse and appraise evidence from all data sources to support decision-making | 1.00 | 5.00 | 4.00 | 1.25 | 9 |
| 2.4 Builds consensus on the key variables and relationships between the variables | 2.00 | 5.00 | 4.22 | 1.03 | 9 |
| 2.5 Applies appropriate model calibration procedures to account for potential biases and uncertainties in the input parameters and calibration targets | 2.00 | 5.00 | 4.44 | 1.07 | 9 |
| 2.6 Applies appropriate sensitivity analysis procedures to understand the effect of implicit and explicit model assumptions | 1.00 | 5.00 | 4.44 | 1.26 | 9 |
| 2.7 Applies appropriate validation procedures with a focus on historical fit | 1.00 | 5.00 | 3.89 | 1.29 | 9 |
| 2.8 Applies appropriate validation procedures with a focus on face validity | 3.00 | 5.00 | 4.33 | 0.82 | 9 |
| 2.9 Translates uncertainty of model outputs | 3.00 | 5.00 | 4.67 | 0.67 | 9 |
| 3.1 Understands the process and aims of evidence-informed decision-making and knowledge transfer in diverse governance contexts | 1.00 | 5.00 | 3.56 | 1.17 | 9 |
| 3.2 Uses the model to evaluate different policy options | 3.00 | 5.00 | 4.00 | 0.67 | 9 |
| 3.3 Identifies the policy implications of the model results | 3.00 | 5.00 | 4.00 | 0.67 | 9 |
| 3.4 Communicates the results effectively within the context of translating science and evidence into practice and policy | 3.00 | 5.00 | 4.00 | 0.94 | 9 |
| 3.5 Assesses when the use of the model beyond the stipulated boundaries (generalisability) is appropriate | 3.00 | 5.00 | 4.11 | 0.74 | 9 |
| 3.6 Enables the use of the model beyond the immediate decision problem if appropriate | 3.00 | 5.00 | 4.11 | 0.74 | 9 |

**Supplementary Table 7 Quantitative analysis of the relevance of the competencies for the Facilitator profile – 1^st^ round.**

| Competency | Minimum | Maximum | Mean | Std Deviation | Count |
| --- | --- | --- | --- | --- | --- |
| 1.1 Demonstrates knowledge of basic business practices, such as terms of reference, business plans, contracting and project management | 3.00 | 4.00 | 3.75 | 0.43 | 4 |
| 1.2 Understands the principles of systems thinking | 4.00 | 5.00 | 4.75 | 0.43 | 4 |
| 1.3 Reflects on the strengths and weaknesses of different modelling methodologies | 4.00 | 4.00 | 4.00 | 0.00 | 4 |
| 1.4 Identifies, connects and manages relationships with stakeholders | 4.00 | 5.00 | 4.50 | 0.50 | 4 |
| 1.5 Builds consensus on the decision problem | 3.00 | 5.00 | 4.50 | 0.87 | 4 |
| 1.6 Builds consensus on the model boundaries | 3.00 | 5.00 | 4.25 | 0.83 | 4 |
| 1.7 Reflects on the role of participatory simulation modelling in diverse governance contexts | 4.00 | 5.00 | 4.75 | 0.43 | 4 |
| 2.1 Reflects on the principles of good dynamic simulation modelling | 2.00 | 5.00 | 3.75 | 1.09 | 4 |
| 2.2 Co-creates conceptual system maps | 3.00 | 5.00 | 4.00 | 0.71 | 4 |
| 2.3 Knows how to retrieve, analyse and appraise evidence from all data sources to support decision-making | 2.00 | 4.00 | 3.50 | 0.87 | 4 |
| 2.4 Builds consensus on the key variables and relationships between the variables | 3.00 | 5.00 | 4.00 | 0.71 | 4 |
| 2.5 Applies appropriate model calibration procedures | 2.00 | 4.00 | 3.00 | 1.00 | 4 |
| 2.6 Applies appropriate sensitivity analysis procedures | 2.00 | 5.00 | 3.25 | 1.30 | 4 |
| 2.7 Applies appropriate validation procedures with a focus on historical fit | 2.00 | 4.00 | 3.00 | 1.00 | 4 |
| 2.8 Applies appropriate validation procedures with a focus on face validity | 2.00 | 4.00 | 3.00 | 1.00 | 4 |
| 3.1 Understands the process and aims of evidence-informed decision-making and knowledge transfer in diverse governance contexts | 4.00 | 5.00 | 4.75 | 0.43 | 4 |
| 3.2 Uses the model to evaluate different policy options | 4.00 | 4.00 | 4.00 | 0.00 | 4 |
| 3.3 Identifies the policy implications of the model results | 4.00 | 5.00 | 4.50 | 0.50 | 4 |
| 3.4 Communicates the results effectively within the context of translating science and evidence into practice and policy | 5.00 | 5.00 | 5.00 | 0.00 | 4 |
| 3.5 Enables the use of the model beyond the immediate decision problem | 2.00 | 4.00 | 3.25 | 0.83 | 4 |

**Supplementary Table 8 Quantitative analysis of the relevance of the competencies for the Facilitator profile – 2^nd^ round.**

| Competency | Minimum | Maximum | Mean | Std Deviation | Count |
| --- | --- | --- | --- | --- | --- |
| 1.1 Understands the principles of systems thinking | 3.00 | 5.00 | 4.44 | 0.83 | 9 |
| 1.2 Reflects on the strengths and weaknesses of different modelling methodologies | 2.00 | 5.00 | 4.00 | 0.94 | 9 |
| 1.3 Identifies, connects and manages relationships with stakeholders throughout the process | 3.00 | 5.00 | 4.78 | 0.63 | 9 |
| 1.4 Builds consensus on the decision problem | 5.00 | 5.00 | 5.00 | 0.00 | 9 |
| 1.5 Builds consensus on the model boundaries | 3.00 | 5.00 | 4.67 | 0.67 | 9 |
| 1.6 Reflects on the role of participatory simulation modelling in diverse governance contexts | 2.00 | 5.00 | 4.22 | 1.03 | 9 |
| 1.7 Applies good analytical and critical thinking | 3.00 | 5.00 | 4.67 | 0.67 | 9 |
| 2.1 Reflects on the principles of good dynamic simulation modelling | 3.00 | 5.00 | 4.11 | 0.74 | 9 |
| 2.2 Co-creates conceptual system maps | 2.00 | 5.00 | 4.33 | 0.94 | 9 |
| 2.3 Knows how to retrieve, analyse and appraise evidence from all data sources to support decision-making | 2.00 | 5.00 | 3.67 | 0.94 | 9 |
| 2.4 Builds consensus on the key variables and relationships between the variables | 3.00 | 5.00 | 4.56 | 0.68 | 9 |
| 2.5 Applies appropriate model calibration procedures to account for potential biases and uncertainties in the input parameters and calibration targets | 1.00 | 5.00 | 2.56 | 1.26 | 9 |
| 2.6 Applies appropriate sensitivity analysis procedures to understand the effect of implicit and explicit model assumptions | 1.00 | 5.00 | 2.89 | 1.45 | 9 |
| 2.7 Applies appropriate validation procedures with a focus on historical fit | 1.00 | 5.00 | 2.67 | 1.25 | 9 |
| 2.8 Applies appropriate validation procedures with a focus on face validity | 2.00 | 5.00 | 3.11 | 1.10 | 9 |
| 2.9 Translates uncertainty of model outputs | 2.00 | 5.00 | 3.89 | 1.10 | 9 |
| 3.1 Understands the process and aims of evidence-informed decision-making and knowledge transfer in diverse governance contexts | 3.00 | 5.00 | 4.33 | 0.67 | 9 |
| 3.2 Uses the model to evaluate different policy options | 2.00 | 5.00 | 4.44 | 0.96 | 9 |
| 3.3 Identifies the policy implications of the model results | 4.00 | 5.00 | 4.78 | 0.42 | 9 |
| 3.4 Communicates the results effectively within the context of translating science and evidence into practice and policy | 4.00 | 5.00 | 4.78 | 0.42 | 9 |
| 3.5 Assesses when the use of the model beyond the stipulated boundaries (generalisability) is appropriate | 1.00 | 5.00 | 4.11 | 1.29 | 9 |
| 3.6 Enables the use of the model beyond the immediate decision problem if appropriate | 3.00 | 5.00 | 4.33 | 0.82 | 9 |
